# Diet quality determines lipase gene expression and lipase/esterase activity in *Daphnia pulex*

**DOI:** 10.1242/bio.022046

**Published:** 2017-01-09

**Authors:** Apostolos-Manuel Koussoroplis, Anke Schwarzenberger, Alexander Wacker

**Affiliations:** Theoretical Aquatic Ecology an Ecophysiology Group, Institute for Biochemistry and Biology, University of Potsdam, 14469 Potsdam, Germany

**Keywords:** Cyanobacteria, Digestive enzyme activity, Nutritional quality, Lipases

## Abstract

We studied the short- (12 h) and long-term (144 h) response of *Daphnia pulex* lipases to quality shifts in diets consisting of different mixtures of the green alga *Scenedesmus* with the cyanobacterium *Synechococcus*, two species with contrasting lipid compositions. The lipase/esterase activity in both the gut and the body tissues had fast responses to the diet shift and increased with higher dietary contributions of *Synechococcus*. When screening the *Daphnia* genome for TAG lipases, we discovered a large gene-family expansion of these enzymes. We used a subset of eight genes for mRNA expression analyses and distinguished between influences of time and diet on the observed gene expression patterns. We identified five diet-responsive lipases of which three showed a sophisticated short- and long-term pattern of expression in response to small changes in food-quality. Furthermore, the gene expression of one of the lipases was strongly correlated to lipase/esterase activity in the gut suggesting its potentially major role in digestion. These findings demonstrate that the lipid-related enzymatic machinery of *D. pulex* is finely tuned to diet and might constitute an important mechanism of physiological adaptation in nutritionally complex environments.

## Abstract (precis)

**Summary:** We show for the first time that the waterflea *Daphnia pulex* has the ability to finely tune its lipolytic machinery to changes in the nutritional quality of its diet.

## INTRODUCTION

*Daphnia* species are filter feeders with a relatively restricted capacity for selecting the food particles they ingest except based on their size (DeMott, 1988). In consequence, the environmental spatiotemporal variability of the biochemical composition of food particles is the main driver of the quality of ingested food. For this key aquatic herbivore, diet quality is defined by the availability of essential biomolecules such as essential fatty acids (FA), sterols (Martin-Creuzburg et al., 2009), and amino acids (Koch et al., 2011), and can be highly variable in time and space. Nutritional variation arises from (1) the strong inherent biochemical differences among ingested food particles (algae, fungi, bacteria, ciliates, detrital particles) (Brett et al., 2009; Colombo et al., 2016; Galloway and Winder, 2015), and (2) the movements of zooplankton between heterogeneous food patches (e.g. diel vertical migration) (Park et al., 2004) and the seasonal succession in the taxonomic composition of phytoplankton and other taxa (Hartwich et al., 2012; McMeans et al., 2015). At the metapopulation scale, *Daphnia* species also experience a pronounced variance in dietary quality and availability due to inter-pond differences (Müller-Navarra et al., 2004). In such a complex dietary environment and without the ability to select ingested particles, the ecological success of a species likely depends on its ability to efficiently adjust the assimilation of food via post-ingestive regulation (Karasov et al., 2011). Recent studies show that *Daphnia* can selectively assimilate nutritional compounds from food (Taipale et al., 2014, 2016), hence suggesting strong post-ingestive regulation abilities.

Among the different mineral and biochemical nutrients composing the diet of *Daphnia*, the lipids are among the most studied and their roles best understood (Arts, 1999; Martin-Creuzburg and von Elert, 2009; Wacker and Martin-Creuzburg, 2007; Müller-Navarra et al., 2004). Lipids are key components of cell membranes, the main energy storage form in *Daphnia*, and are heavily invested in eggs (Arts, 1999). The extraction and uptake of dietary lipids depends on carboxyl ester hydrolases (E.C. 3.1.1) and triacylglycerol (TAG) lipases. Lipases are also responsible for mobilization, routing and metabolism of internal lipid stores. Hence, lipases play an essential role in energetic and structural homeostasis and constitute an important regulatory interface between consumers and their food (Horne et al., 2009). Nevertheless, *Daphnia* lipases have remained virtually unstudied since Hassler (1935) detected lipolytic activity in *Daphnia* guts. Previous studies on marine copepods found species- and context-specific digestive enzyme activity responses to changes in food quality and quantity, in some cases within less than 24 h (Guérin and Kerambrun, 1982; Kerambrun and Champalbert, 1993; Kreibich et al., 2011). Changes in digestive enzymatic activity reflect quantitative and/or qualitative modifications of the enzyme pool and they are considered as an important mechanism of nutrient uptake regulation and adaptation to dietary variance (Clissold et al., 2010; Karasov et al., 2011).

Generally speaking, there are two opposing views on how digestive enzymes respond to diet quality shifts. The first is that the consumer should always maximize the extraction of the nutrients from food and then use post-absorptive mechanisms to regulate the retention of nutrients. Hence, digestive enzyme secretion should vary positively with the substrate concentration in diet (Caviedes-Vidal et al., 2000; Raubenheimer and Bassil, 2007). The second view states that consumers also regulate the make-up of absorbed nutrients by maximizing the extraction of the most limiting nutrient in the diet. Thus, the secretion of digestive enzymes should vary homeostatically, i.e. with enzymes for nutrients in excess being secreted at lower rates than enzymes for nutrients in deficit (Bansemer et al., 2016; Clissold et al., 2010).

We investigated the short- (12 h) and longer-term (6 days) responses of the lipid metabolism of *D.* *pulex* to diet in a food switch experiment following two lines of research. The changes in the total lipase/esterase activity and the changes in expression of a group of triacylglycerol (TAG)-lipase/steryl-esterase genes. In nature, green algae and cyanobacteria contribute to a large extent to the material ingested by *Daphnia*, and cyanobacteria generally reduce *Daphnia* growth and reproduction (Lukas and Wacker, 2014a; Wacker and Martin-Creuzburg, 2007). From a lipid nutrition perspective, cyanobacterial lipids strongly differ, both quantitatively and qualitatively: (1) chlorophyta lipids account for 23% of dry weight, whereas cyanobacterial dry weight consists of only 8% lipids; (2) chlorophyta membranes mainly contain phosphatidylcholines, whereas cyanobacterial membranes are rich in phosphatidylglycerol; and (3) cyanobacterial lipids are typically deficient in highly-unsaturated fatty acids (HUFA) and sterols, both essential for *Daphnia* (Griffiths and Harrison, 2009; Harwood, 1998; Martin-Creuzburg et al., 2008; Murata and Nishida, 1987; Wacker and von Elert, 2001). In this experiment, *Daphnia* acclimated to a pure green algal diet (*Scenedesmus obliquus*) were shifted to a set of diets containing gradually increased proportions of a cyanobacterium (*Synechococcus elongatus*). We hypothesized that if responses of the lipid metabolism to the diet occur, they should match the dietary gradient in the cyanobacterium proportion.

## RESULTS

### Lipase/esterase activity

**Fig. 1.**
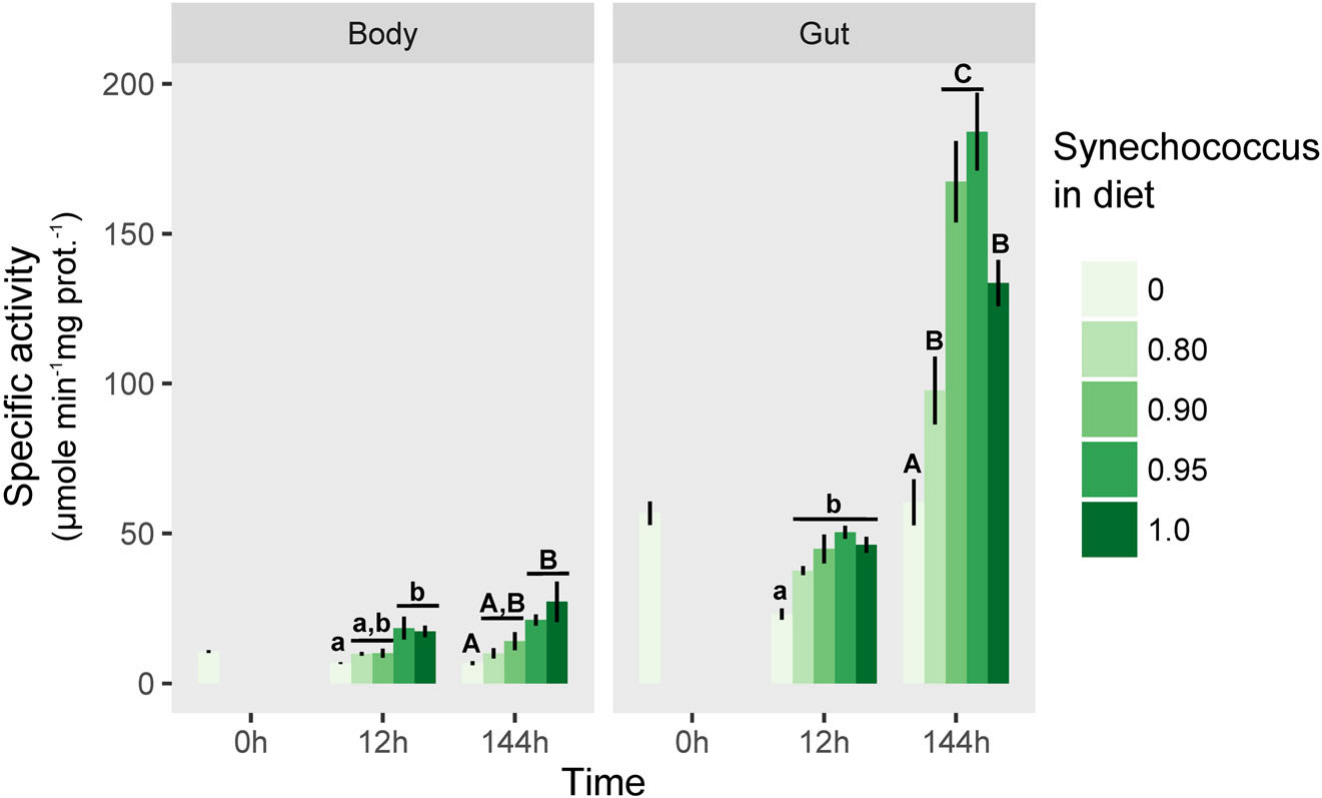
**Lipase/esterase specific activity in *Daphnia pulex* tissues.** Lipase/esterase specific activity (µmol min^−1^ mg total protein^−1^; mean±s.d., *n*=3) in the body (left panel) and the gut tissues (right panel) measured after switching from a pure *Scenedesmus obliquus* diet to diets containing different proportions of *Synechococcus elongatus*. Different lower case or capital letters indicate significant differences within their respective time scales (one-way ANOVA, followed by Tukey HSD post hoc test, *P*<0.05).

In the gut, diet significantly affected lipase activity at both time scales (12 h: ANOVA, *F*_4,15_=14.23, *P*<0.0001; 144 h: ANOVA, *F*_4,15_=23.27, *P*<0.0001) with activities generally increasing with even slight increases in the proportion of *S. elongatus* in the diet mixture (Fig. 1). Indeed, raising the proportion of *S. elongatus* from 80 to 90-95% led to a nearly twofold increase in lipase activity (Fig. 1). However, the lipase activity on pure *S. elongatus* diet decreased again to that of the 80% *S. elongatus* level (Fig. 1). The lipase activity pattern was similar in the body, although the lipases showed much lower activity levels than in the gut (12 h: ANOVA, *F*_4,15_=6.23, *P*<0.001; 144 h: ANOVA, *F*_4,15_=5.72, *P*=0.011; Fig. 1).

### Lipase gene expression analysis

For gene-expression analyses we used whole-body homogenates of *Daphnia*. Since we found a similar pattern of lipase activity for the body and for the gut, we were certain that gene expression would also show a similar pattern for both sample types. Also, since lipase activity was 10-times higher in the *Daphnia* gut than in the body, the gene expression in the gut should project above gene expression in the body and should therefore play a minor role in qPCR analyses.

**Fig. 2.**
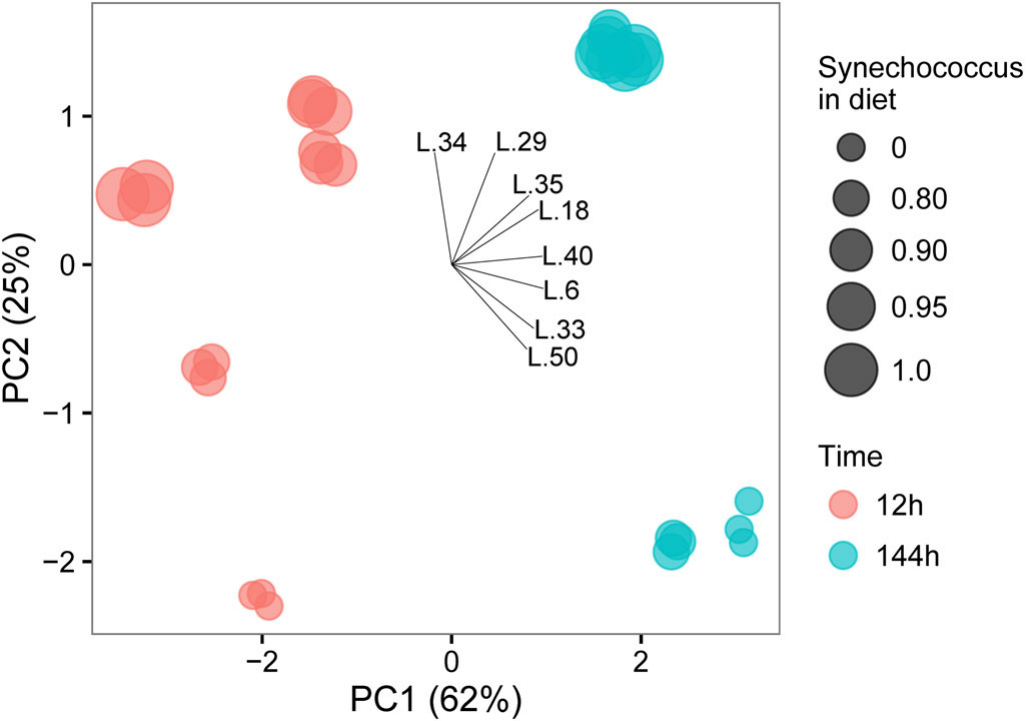
**Principal component analysis (PCA) biplot of lipase gene expression in *Daphnia pulex*.** The PCA of the different temporal and diet treatments is based on the relative expression of the lipase genes (L._x_) in *Daphnia pulex* measured 12 h and 144 h after switching from a pure *Scenedesmus obliquus* diet to diets containing different proportions of *Synechococcus elongatus*. The relative gene expression is measured as the log_2_ fold increase relative to that for *Scenedesmus obliquus* before the diet switch (i.e. 0 h). The amount of variance explained by each principal component axis is shown in parentheses. Each depicted datum is a biological replicate.

The two first principal components (PC) explained 87% of the variation in the gene expression data. PC1 explained 62% of the variation and separated the two temporal treatments. 144 h samples had higher PC1 scores indicating high expression for L40, L18, L6, L35, L33 and to a lesser extent L29 (Fig. 2). The diet treatments were separated on the PC2 which explained 25% of the variation. The treatment switched to diets with lower proportions of *S. obliquus* had higher PC2 scores associated with higher expression for L34, L29, L35, and L18, and lower L50, L33, and L6 expressions (Fig. 2). We could thus clearly identify those genes mostly responding to diet (L34, L29, L33, L50, L35) and those that were less (L6, L18,) or not at all (L40) influenced by food changes.

**Fig. 3.**
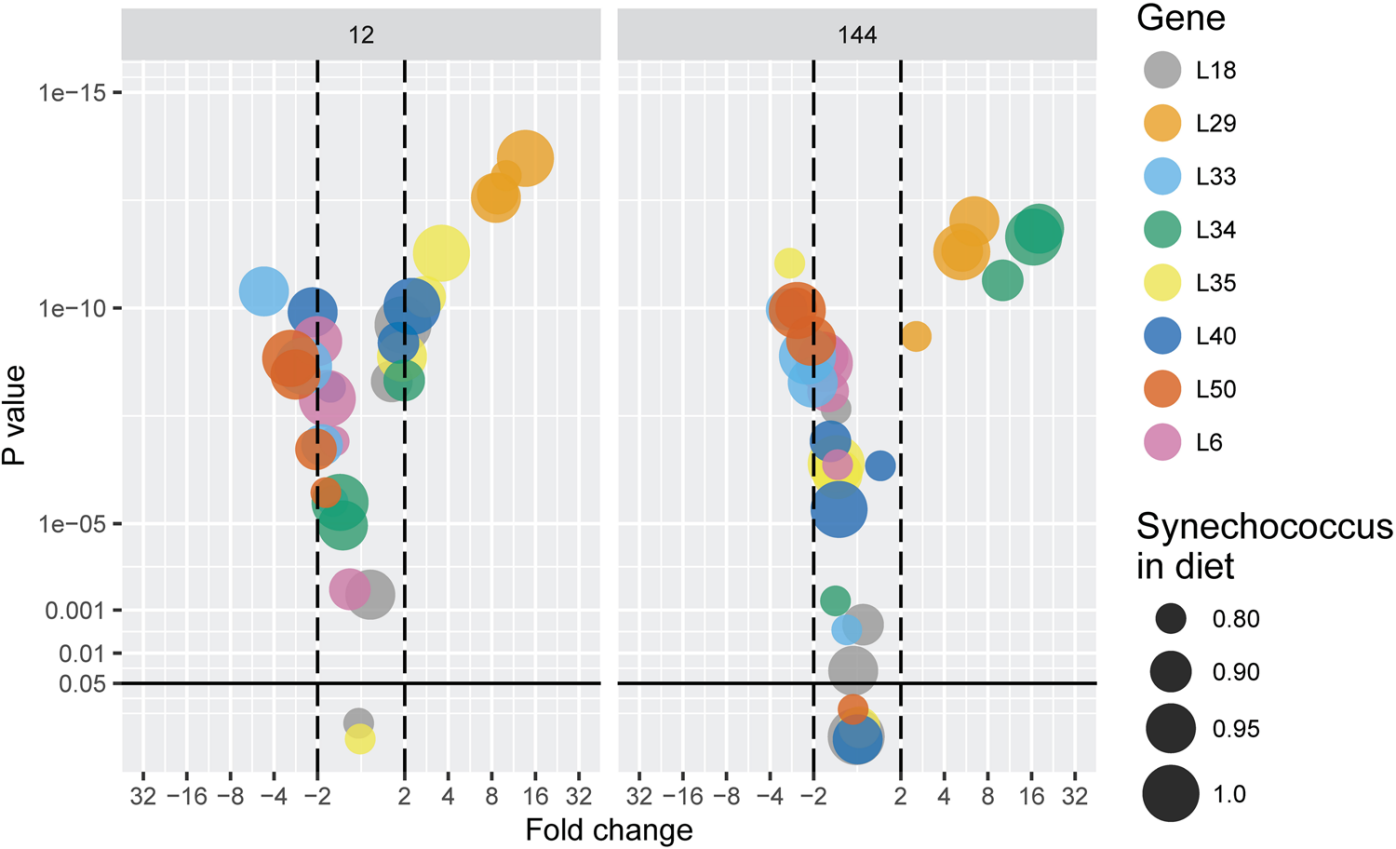
**Volcano plot analysis of the lipase relative expression in *Daphnia pulex*.** Gene expression (mean, *n*=3) is measured (left panel) 12 h and (right panel) 144 h in *Daphnia pulex* after switching from a pure *Scenedesmus obliquus* diet to diets containing different proportions of *Synechococcus elongatus*. Each gene is depicted by a color and the size of the circles indicates the relative proportion (in terms of carbon) of *S. elongatus* in the new diet (0 indicates a pure *S. obliquus* control). The horizontal axis expresses the biological significance of the changes in gene expression. Values are fold change (log_2_ axis scaling) in expression relative to the *S. obliquus* control*.* Positive values indicate up-regulation while negative values down-regulation. Values outside the area delimited by the vertical dashed lines changes are considered biologically significant. The vertical axis expresses the statistical significance of the change estimated by the *P*-values (-log_10_ axis scaling) of the comparisons between diet treatments and the pure *S. obliquus* control (Tukey HSD post hoc test, see Materials and Methods). The solid horizontal line indicates the statistical significance threshold (*P*=0.05). *See* also Fig. S2 for single gene plots.

After 12 h, the changes were both statistically and biologically significant (more than twofold increase) for L33 and L50 (decrease) as well as for L35 and L29 (increase) (Fig. 3; *see* also Fig. S2 for single gene plots). For L29 the increase in expression was more than tenfold higher for most of the *S. elongatus* treatments. The decrease in L33 and L50 expressions was gradual and negatively correlated to the proportion of *S. elongatus* in the diet up to 95% where it appeared to level-off (Fig. 3). The increase in the expressions of L29 and L35 occurred between 80 and 90% of *S. elongatus* without further changes **(**Fig. 3**)**. After 144 h, biologically significant responses were mostly observed for L33 and L50 (decrease) as well as L34 and L29 (increase) (Fig. 3). The decrease in L33 and L50 with increasing *S. elongatus* was gradual (Fig. 3). The increase in the expressions of L29 and L34 (more than tenfold) was also gradual and tended to level-off above 90% of *S. elongatus* in diet (Fig. 3).

## DISCUSSION

As filter feeders, *Daphnia* have limited control over the food they ingest, thereby being exposed to fluctuating dietary lipid quantity and quality in their gut contents across various temporal scales. Here, we study two ecologically relevant scales for *Daphnia*: the sub-daily scale (12 h) which reflects fast diet changes due to vertical or horizontal diurnal movements between microhabitats that might differ in their food sources; and the weekly scale (144 h), which reflects the slower dietary lipid fluctuations such as those driven by phytoplankton species temporal succession. Our results indicate that *D. pulex* is able to respond to such changes by controlling the lipase/esterase activity in their gut. More specifically, *D. pulex* responded to the cyanobacterial diet by increasing the lipase/esterase activity both in the gut and body tissue. The response was consistent across time scales, and the magnitude matched, as hypothesized, the proportion of the lipid-poor cyanobacteria in the diet.

Given the lower lipid contents of the cyanobacterium (*S. elongatus)* as compared to green alga (*S. obliquus*) and its lack in essential lipids (i.e. HUFA and sterols) (Griffiths and Harrison, 2009; Harwood, 1998; Martin-Creuzburg et al., 2008; Murata and Nishida, 1987; Wacker and von Elert, 2001), the observed pattern points towards a homeostatic digestive enzyme secretion that could aim to maximize the extraction of the most limiting nutrient in the diet (Bansemer et al., 2016; Clissold et al., 2010). Hence, increased lipase/esterase activities could compensate for a decreasing supply in essential HUFA (or sterols) by promoting the extraction efficiency of these molecules from gut contents. This could be in agreement with observed reduction of ingestion rates of *Daphnia* switched from *S. obliquus* to *S. elongatus* (Lukas and Wacker, 2014b), which could aim to prolong the gut passage time and further increase the extraction efficiency of lipids (Karasov et al., 2011). In *Daphnia*, such responses have also been observed for alkaline phosphatase activity (Elser et al., 2010; McCarthy et al., 2010; Wagner and Frost, 2012; Wojewodzic et al., 2011) which increased when the phosphorus content of the diet decreased.

In addition to the differences in lipid content, the differences between the FA compositions *S. obliquus* to *S. elongatus* lipids could also influence the digestibility of lipids per se, thereby inducing different lipase/esterase activity responses. In fish, the digestibility of TAG has been shown to decrease with increasing chain length and to increase with unsaturation (Morais et al., 2007 and references therein), and a similar substrate-specificity pattern has been observed for the lipolysis of stored TAG in mammalian tissues (Raclot, 2003). Hence, changes in lipase/esterase activity could reflect the overall digestibility of TAG. In our case however, the switch to a cyanobacteria-dominated diet implies both a decrease in FA chain length and a decrease in FA unsaturation. These concomitant changes are expected to affect TAG digestibility in opposed directions (increase and decrease of digestion efficiency, respectively). Hence, it is unclear whether the net effect of the diet switch on TAG digestibility is high enough to induce an increase in lipase/esterase secretion. The single molecule (MUB) used in our assays is highly digestible, and does not allow us to specifically address such issues, but future studies could explore it by measuring lipolytic activity on various substrates.

The lipolytic activity pattern in body tissue was similar to that in the gut, although the lipases/esterases showed much lower activity levels. This result indicates that a series of post-assimilation processes are set in motion after the diet quality switch in order to regulate the structural and/or energetic homeostasis. For example, in order to face the decreased supply in HUFA and cholesterol, *Daphnia* exposed to *S. elongatus*-rich diets might be increasing the secretion of TAG-lipases/steryl-esterases in the body tissues in order to accelerate the break-down of their lipid reserves. This could allow mobilizing and routing stored HUFA and cholesterol towards cell membranes or reproductive tissues where these lipids play a major role; this observation could be also due to increased phospholipase activity. Certain phospholipases are involved in the repair of cell membranes submitted to oxidative damage (Horne et al., 2009). *Daphnia* fed cyanobacterial diets exhibit increased respiration rates (Lukas and Wacker, 2014a), a situation associated with increased oxidative stress (Steinberg et al., 2010).

### TAG-lipase gene expression patterns

The changes in lipase/esterase activity can either derive from an increase in the amount of enzymes (i.e. increase of mRNA levels or the recruitment of additional isoforms) or the switch in composition of lipase pool. Our results show a large gene-family expansion of TAG-lipases/steryl-esterases which grouped in four different clusters in the *D. pulex* phylogenetic tree. This observation is in line with the generally high number of expanded gene-families in *Daphnia* (Colbourne et al., 2011) and the surprisingly high levels of variation within functionally identical gene-families within a single clone (Schwarzenberger et al., 2009; Schwerin et al., 2009). Such gene family expansions are considered as an element of the success of *Daphnia* in diverse and variable environments (Colbourne et al., 2011).

Within the subset of the studied lipases we found five out of the eight studied genes responded to diet shifts in a biologically significant manner [i.e. more than twofold down- (L50, L33) or up-regulation (L29, L34, L35)]. Furthermore, expression patterns showed some evidence for differential short- and long-term regulation. L35 seems to be only involved in short-term responses whereas L34 only responds to diet-shifts after 144 h. Interestingly, as for the lipase/esterase activity, the magnitude of the expression changes matched the experimental dietary gradient, further suggesting that *D. pulex* restructures its lipase enzymatic machinery as a response to some quantifiable food quality trait. The most interesting response to changes in diet quality showed L29 (a putative triglyceride lipase-cholesterol esterase), which was the only lipase whose gene expression was up-regulated both after short- and long-term exposure. Additionally, the gene expression levels of L29 were strongly positively correlated to the changes in gut lipase/esterase activity independently of whether 12 h, 144 h or all samples were considered (r²=0.73-0.86, *P*<0.0001; Fig. S1). Since it is a rare phenomenon that a clear correlation between gene expression and activity of an enzyme can be observed, this finding warrants further research to verify the hypothesis that L29 could be coding for a major digestive lipase in *D. pulex*.

By dynamically adjusting the substrate-specificity of the digestive lipases pool, daphnids could be able to maintain high lipid extraction efficiency under a fluctuating supply of substrate quality. For example, the marine copepods *T. longicornis* exposed to various diet-switches between different algal species showed marked and reversible changes in the composition of their lipase/esterase isoenzyme pool within few hours (Kreibich et al., 2011). We conclude that the activity changes of the gut lipases/esterases could be due to a combined response of several genes. Such a complex gene expression/activity pattern might reflect *Daphnia*'s necessity to maintain all members of such a huge gene-family expansion. Clearly, more genes (including phospholipase genes) need to be investigated in future studies to obtain the complete picture and test the above-mentioned hypotheses.

Our study indicates that the regulation of lipase/esterase activity in the gut is a potentially important physiological mechanism employed by daphnids in order to buffer variability in dietary lipid supply. Furthermore, our results show that the response to diet quality changes include a complex expression regulation of a highly expanded lipase gene-family. This suggests that the substrate specificity of the TAG-lipases/steryl-esterases might be also adjusted to the diet. Most importantly, our results show that *D. pulex* is not only responding to the shift in diet quality but also to the amount of change. This indicates that the response is regulated by the availability of certain compounds in the diet. Whether these compounds are FA in general, sterols, or specific HUFA remains to be determined. Given the major role of lipids as regulators of trophic flows at the aquatic plant-animal interface, our results indicate promising perspectives for studying zooplankton-phytoplankton interactions in nature.

## MATERIALS AND METHODS

### Experimental design and settings

Monoclonal neonates of *D. pulex* hatched within 12 h were kept in filtered and aerated lake water vessels containing 2 mg C L^−1^ of *S. obliquus* as food, renewed every other day. We used a clone of *Daphnia pulex* (clone Münster) isolated from a pond in Gievenbeck (51°57′47,16 N, 7°34′38,09 E), Germany*.* The green alga *Scenedesmus obliquus* (SAG 276-3a, culture collection of algae, University of Göttingen, Göttingen, Germany) and the cyanobacterium *Synechococcus elongatus* (SAG 89.79). *S. elongatus* and *S. obliquus* were cultured semi-continuously (dilution rates: 0.2 d^−1^ and 0.4 d^−1^, respectively) at a light:dark cycle of 16 h:8 h in aerated 2 liter flasks using WC medium (Guillard and Lorenzen, 1972) with vitamins and illumination at 40 µmol m^−2^ s^−1^ and 120 µmol m^−2^ s^−1^, respectively. The estimation and adjustment of carbon concentration in the dietary treatments was based on the measurements of the optical density of phytoplankton and carbon–light extinction regression established prior to the experiment for each algae (Sperfeld et al., 2012).

The diet shift experiment was initiated once the daphnids reached a sufficient size for gut dissection, i.e. 3 weeks old. At the onset of the experiment, a triplicate sample of *Daphnia* was taken for gene expression and enzymatic activity analyses. The remaining daphnids were randomly distributed in 1 liter glass vials (∼15 individuals vial^−1^) with five different food suspensions including three mixtures of *S. elongatus* with *S. obliquus* (20%, 10% and 5% of *S. elongatus*), 100% *S. elongatus* and 100% *S. obliquus* (as reference treatment). All food treatments were replicated six times. The food suspensions were renewed daily and the total algal concentration was adjusted to 2 mg C L^−1^. At each sampling time (12 and 144 h after the diet shift), three replicate food treatments were sacrificed for lipase gene expression and enzymatic activity analyses.

From each sacrificed replicate four individuals were pooled and subsequently used for gene expression, immediately flash-frozen in liquid nitrogen and stored at −80°C until RNA extraction. The remaining individuals (*n*=11) were dissected under a binocular microscope and the whole gut, including the hepatopancreas, was separated from the rest of the body. The animals were put on a microscope slide on top of a frozen thermal pack in order to prevent immediate death of the animal and degradation of gut enzymes. Then, the carapax was steadied with a forceps while the head together with the hepatopancreas and the intact gut were torn out with a second set of forceps. Individual guts or bodies from the same replicate were pooled, respectively, and homogenized in ice-cold 150 µl Tris/HCl buffer (0.1 M, pH 7.5). The extracts were centrifuged for 10 min (15,000 ***g***, 4°C). The supernatant was split for ulterior total soluble protein and lipase activity analyses and immediately flash-frozen in liquid nitrogen. Samples were stored at −80°C and processed within few days.

### Total soluble protein and lipase/esterase activity measurements

Water soluble protein content of the guts and body tissues of *D. pulex* was measured in microplates with the bicinchoninic acid assay (BCA kit, Pierce Ltd.) following manufacturer's instructions. The microplates were read at 550 nm with a microplate reader (Infinite F200 Pro, TECAN^®^, Männedorf, Switzerland). Bovine serum albumin (BSA, 1 to 5 μg per well) was used as standard.

The lipase/esterase activity [carboxylic ester hydrolases (E.C. 3.1.1)] in the guts and body tissues of *D. pulex* was determined fluorometrically using substrate 4-methylumbelliferyl butyrate (MUB). The supernatants were further diluted twice in Tris/HCl buffer (0.1 M, pH 7.5). 25 µl of the samples were incubated in microplate wells with a 275 µl of MUB solution (final MUB concentration in well: 100 µM). Fluorescence was measured at 360 nm (excitation) and 450 nm (emission) after 2.5, 5, 7.5, 10, 15, 20, 30 and 45 min (Infinite F200 Pro, TECAN^®^). The activity was determined using the linear phase of fluorescence increase. Standard curves were prepared with 4-methylumbelliferone. The rate of MUB autolysis was determined and subtracted from the results. Enzyme activities were expressed in nmol min^−1^ mg protein^−1^.

### RNA extraction and qPCR

RNA was extracted from each replicate with the NucleoSpin RNA Kit (Macherey and Nagel, Düren, Germany). The animals were homogenized with a pestle in 350 µl RA1. 350 µl, 70% ethanol was added and the mixture was mixed before loading onto an RNA binding column. The column was centrifuged (11,000 ***g*** 30 s) and desalted with 350 µl MDB at 11,000 ***g*** for 1 min. The RNA was incubated with 95 µl DNase reaction mixture for 20 min in order to digest genomic DNA. Afterwards the RNA was washed with RAW2 and RA3 as according to the manufacturer's protocol. RNA was eluted with 60 µl RNase-free water. RNA was reverse transcribed with High-capacity cDNA Reverse Transcription Kit (Life Technologies, Darmstadt, Germany). The integrity of the RNA was verified with 1.5% agarose gel electrophoreses. RNA concentrations were determined with a Qubit fluorometer (Invitrogen) as per the manufacturer's instructions.

qPCR and data analyses were performed as according to Schwarzenberger et al. (2009). qPCR was conducted on the 7300 real time PCR system (Applied Biosystems, Thermo Fisher Scientific, Schwerte, Germany). Each reaction contained 3 ng of cDNA template, 10 µl Power SYBR^®^ Green PCR Master Mix (Life Technologies, Darmstadt, Germany) and 2.5 µM of each primer (Table 1) in a final volume of 20 µl. Each reaction was conducted in three biological replicates. Cycling parameters were 95°C for 10 min to activate the DNA polymerase followed by 40 cycles of 95°C for 15 s and 60°C for 1 min. After the actual analysis, dissociation curves were performed to verify that no primer-dimers had been amplified.

**Table 1.**
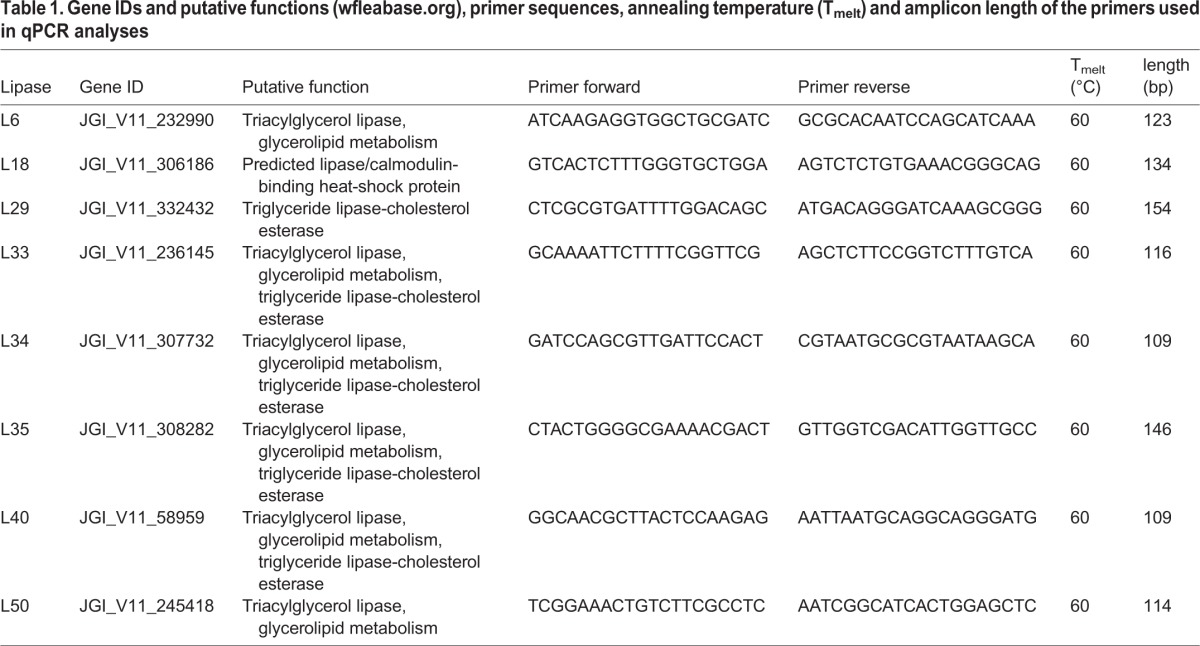
**Gene IDs and putative functions (wfleabase.org), primer sequences, annealing temperature (T_melt_) and amplicon length of the primers used in qPCR analyses**

### Phylogenetic tree construction and target gene selection

**Fig. 4.**
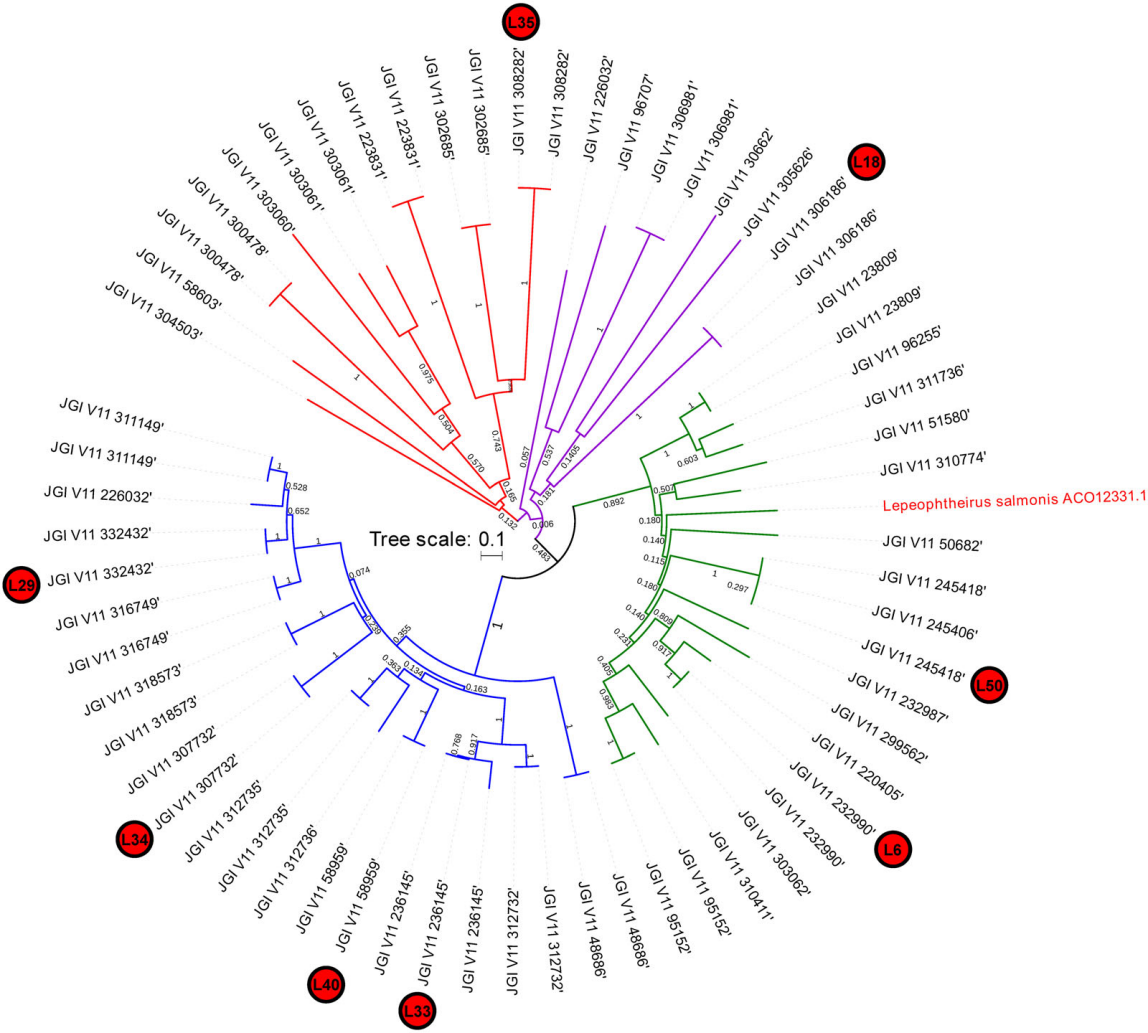
**Neighbor-joining phylogenetic tree of *Daphnia* TAG-lipases.** The eight lipase sequences chosen for primer development and expression as well as the outgroup sequence are indicated.

For normalization, three different endogenous controls [18S ribosomal RNA (18S), *Tryptophanyl- tRNAsynthetase* (WARS), ubiquitin-conjugating enzyme (UBC)] were used in qPCR analysis (Heckmann et al., 2006). We found 63 lipase genes and/or gene copies which grouped in four different clusters in the *D. pulex* phylogenetic tree (Fig. 4) with the pancreatic lipase of the crustacean *L. salmonis* serving as the outgroup. The tree was constructed with Mega 6.0 (Tamura et al., 2013) using the Poisson model (500 bootstrap replicates) with Bootstrap Test of Phylogeny. For this, the exons of 63 lipase gene sequences from the *D. pulex* genome database (Colbourne et al., 2005) were translated into amino acid sequences and compared with the protein sequence of a pancreatic TAG lipase precursor from *Lepeophtheirus salmonis* (NCBI accession number ACO1233.1). Two genes clustering with the outgroup's pancreatic lipase were chosen for gene-expression analysis. Since we also found TAG in the other clusters (Table S1) we chose six further lipases homogeneously distributed along the other three clusters for subsequent gene expression analyses. Eight primer pairs for lipase genes were used in qPCR analysis (Table 1). After determination of the normalization factor with Genorm, which is embedded in qbase^PLUS^ 2.0 (Biogazelle, Zwijnaarde, Belgium; http://www.qbaseplus.com), the relative expression of the target genes was calculated. For each gene, the samples from the start point (0 h, 100% *S. obliquus*) served as calibrator for relative gene expression and were always set as 1.

### Data analysis

The effects of diet was separately evaluated for 12 h and 144 h using in all the cases a one-way ANOVA followed by a Tukey HSD post hoc test. The patterns of expression of the selected genes across the different treatments were explored by principal component analysis (PCA) based on a correlation matrix and log transformed data. The volcano plot approach was used as diagnostic for the combined statistical and biological significance of the gene expression changes. For that, the gene expression was converted to the logarithm (log_2_) of the fold change between expression in a given treatment and in the 100% *S. obliquus* control treatment*.* The statistical significance of the differences between treatments and the 100% *S. obliquus* control was also evaluated at each time scale by one-way ANOVA followed by a Tukey HSD post hoc test. The *P*-values of the Tukey-HSD tests were then log_10_-transformed and incorporated into the plot. The significance level for all tests was set to *P*<0.05.
